# Thickness-Dependent Evolutions of Surface Reconstruction and Band Structures in Epitaxial *β–In_2_Se_3_* Thin Films

**DOI:** 10.3390/nano13091533

**Published:** 2023-05-03

**Authors:** Qinghao Meng, Fan Yu, Gan Liu, Junyu Zong, Qichao Tian, Kaili Wang, Xiaodong Qiu, Can Wang, Xiaoxiang Xi, Yi Zhang

**Affiliations:** 1 National Laboratory of Solid State Microstructure, School of Physics, Nanjing University, Nanjing 210093, China; 2 Collaborative Innovation Center of Advanced Microstructures, Nanjing University, Nanjing 210093, China; 3 School of Physics and Electronic Sciences, Changsha University of Science and Technology, Changsha 410114, China

**Keywords:** In_2_Se_3_, electronic structure, surface reconstruction, molecular beam epitaxy, angle-resolved photoemission spectroscopy

## Abstract

Ferroelectric materials have received great attention in the field of data storage, benefiting from their exotic transport properties. Among these materials, the two-dimensional (2D) In_2_Se_3_ has been of particular interest because of its ability to exhibit both in-plane and out-of-plane ferroelectricity. In this article, we realized the molecular beam epitaxial (MBE) growth of *β–In_2_Se_3_* films on bilayer graphene (BLG) substrates with precisely controlled thickness. Combining in situ scanning tunneling microscopy (STM) and angle-resolved photoemission spectroscopy (ARPES) measurements, we found that the four-monolayer *β–In_2_Se_3_* is a semiconductor with a (9 × 1) reconstructed superlattice. In contrast, the monolayer *β–In_2_Se_3_*/BLG heterostructure does not show any surface reconstruction due to the interfacial interaction and moiré superlattice, which instead results in a folding Dirac cone at the center of the Brillouin zone. In addition, we found that the band gap of In_2_Se_3_ film decreases after potassium doping on its surface, and the valence band maximum also shifts in momentum after surface potassium doping. The successful growth of high-quality *β–In_2_Se_3_* thin films would be a new platform for studying the 2D ferroelectric heterostructures and devices. The experimental results on the surface reconstruction and band structures also provide important information on the quantum confinement and interfacial effects in the epitaxial *β–In_2_Se_3_* films.

## 1. Introduction

In recent decades, the research on ferroelectric materials, which have spontaneous electric polarization, has led to the development of data storage devices [1,2,3,4], such as ferroelectric field effect transistors (FeFETs) and non-volatile memory technology [1,2,5,6,7,8]. However, traditional ferroelectric materials, such as perovskite compounds, usually have only one direction of dipole due to the non-centrosymmetric charge distribution in their crystal, which limits their practical applications [9,10]. As a two-dimensional (2D) van der Waals (vdW) material, In_2_Se_3_ is theoretically predicted to exhibit both in-plane and out-of-plane ferroelectricity when in the monolayer (ML) limit [11,12]. On the other hand, 2D vdW materials, such as graphene, have excellent properties, such as ease of mechanical exfoliation and high in-plane carrier mobility, making them suitable for the fabrication of nano-scale devices [13,14]. Many types of 2D materials with novel properties have been fabricated by either mechanical exfoliation or epitaxial growth methods [15,16]. These materials are well-suitable for building heterostructures and magic-angle systems [17,18]; therefore, they have been used to explore novel quantum states, such as topological [19,20] and superconducting phases [21,22]. The excellent properties of 2D heterostructures also provide them great application potentials in electronic devices [23,24,25]. In addition, the band structure of some 2D materials, such as MoSe_2_ and WSe_2_, undergoes a layer-dependent transition from a direct to an indirect bandgap semiconductor [26,27], and similar thickness-dependent band evolution has also been observed in the Ⅲ–Ⅵ vdW materials, such as InSe and In_2_Se_3_ [28,29,30,31].

Both the lattices of ML *α-*In_2_Se_3_ and *β–*In_2_Se_3_ consist of a five-atomic-layer structure of Se-In-Se-In-Se but showing different stacking orders [32]. The room-temperature ferroelectricity of *α–*In_2_Se_3_ can lead to the characteristic butterfly-like traces of electronic transport curves in the *α–*In_2_Se_3-_based 2D FeFET devices [33,34,35,36,37]. Furthermore, due to the built-in interfacial electric field, the electronic structures of In_2_Se_3_-based heterostructures can be modified depending on which side of the ferroelectric layer is in contact with other 2D materials [11]. In addition to its ferroelectric properties, the optoelectronic and thermoelectric properties of 2D In_2_Se_3_ also broaden its application potentials in the related fields [38,39,40,41,42]. However, unlike the transition metal dichalcogenides (TMDCs), Ⅲ_2_–Ⅵ_3_ compounds usually have complex phase diagrams, including the 2D vdW *α* and *β* phases, and the 3D compound *γ* phase [43], making it difficult to synthesize In_2_Se_3_ with precise phase control [35,44,45,46,47,48,49,50,51].

In this article, by using the molecular beam epitaxial (MBE) method with *α–In2Se3* and Se shots as the evaporation sources, we realized the growth of pure-phase *β–In_2_Se_3_* films on bilayer graphene (BLG)-terminated SiC substrate in ML limit. Using in situ scanning tunneling microscopy (STM), we found that the grown ML *β–*In_2_Se_3_/BLG heterostructure shows a moiré superlattice, while the 4 ML *β–*In_2_Se_3_ film shows a (9 × 1) reconstructed superlattice. The ex situ Raman spectra confirmed the pure *β* phase of the grown In_2_Se_3_ films, although the evaporation source materials were in *α* phase. By combining the in situ X-ray photoemission spectroscopy (XPS) and angle-resolved photoemission spectroscopy (ARPES), we studied the electronic structures of both pristine and potassium-doped *β–*In_2_Se_3_ films. The grown *β–*In_2_Se_3_ films are semi-conductive with an indirect band gap, but the moiré superlattice of ML *β–*In_2_Se_3_/BLG heterostructure induces the Dirac cone folding of BLG substrate. Aside from the energy shifts and gap shrinkage of the band structures, the surface potassium doping on the *β–*In_2_Se_3_ surface also changes the momentum position of valence band maximum (VBM) in reciprocal space.

## 2. Methods

The growth of In_2_Se_3_ films was performed in a combined MBE-STM-ARPES ultra-high vacuum (UHV) system with a base pressure of ~1.5 × 10^−10^ mbar. The BLG substrates were prepared by flash-annealing 4H-SiC(0001) wafers to 1250 °C for 80 cycles [52,53]. High-purity Se (99.9995%) and *α–*In_2_Se_3_ (99.999%) shots served as the constituents to grow In_2_Se_3_ films and were evaporated separately from standard Knudsen cells at 120 °C and 640 °C, respectively. During the growth, the BLG substrate was kept at 300 ℃, and the flux ratio of *α–*In_2_Se_3_ to Se was kept at ~1:8. The growth rate of *β–*In_2_Se_3_ film was ~4 min per ML. The surface morphology was characterized by the in situ reflection high-energy electron diffraction (RHEED) and room-temperature (RT) STM. The RT-STM is a Pan-style one (GC Innovation (Changzhou) Co., Ltd., Changzhou, China), and the tungsten tips for STM measurements were prepared by electrochemical corrosion method. The energy of electron beam for RHEED was set as 15.0 keV. The ex situ Raman scattering spectroscopy was performed with 532 nm laser excitation. The Raman signal was collected using a grating spectrograph and a liquid-nitrogen-cooled charge-coupled device. The sample was mounted in a vacuum chamber during Raman data acquisition. The incident laser was focused on the sample to a micron-sized spot, and the scattered light was detected through Bragg notch filters to access the low-wave-number region. The in situ XPS and ARPES spectra were collected by a DA30 analyzer bought from Scienta Omicron AB, Uppsala, Sweden. The monochromatic X-ray was generated from an Al electrode excitation source (Alα, 1486.7 eV), and the ultraviolet light was generated from a helium lamp (Fermion Instruments (Shanghai) Co., Ltd., China) with a monochromator (He I, 21.218 eV). During the XPS and ARPES measurements, the sample was cooled down to ~8 K by a helium-free close-cycle cryo-manipulator. The potassium doping was conducted in situ by an alkali metal dispenser bought from SAES Getters, S.p.A, Milan, Italy. The heating current of 5.20 A was applied to the potassium dispenser for 40 min for all the surface doping operations. During the potassium doping, the temperature of sample was kept at 8 K, and the doped potassium adatoms are suggested in a disordered arrangement [54].

## 3. Results and Discussion

### 3.1. Surface Reconstructions of the Grown In_2_Se_3_ Films

**Figure 1:**
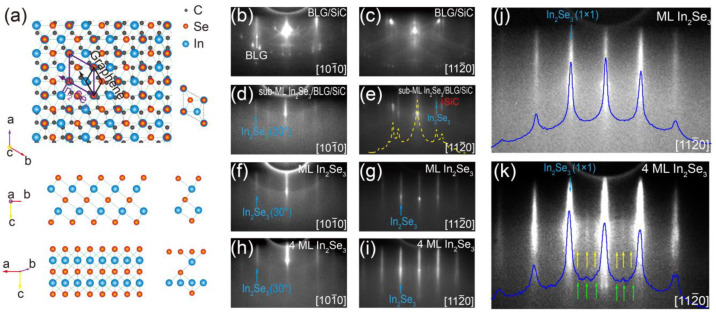
(**a**) Top view (upper panel) and side views (middle and lower panels) of the ML *β–In_2_Se_3_* lattice on BLG. The purple and black rhombuses represent the unit cell of *β–In_2_Se_3_* and the graphene lattice, respectively. The corresponding views of the *β–In_2_Se_3_* unit cell from three directions are shown on the right. (**b**,**c**) RHEED patterns of a BLG/SiC substrate along the (**b**) $\left[10\bar{1}0\right]$ and (**c**) $\left[11\bar{2}0\right]$ directions of SiC, respectively. (**d**–**i**) RHEED patterns for (**d**,**e**) a partially covered sub-ML *β–In_2_Se_3_* film, (**f**,**g**) a fully covered ML *β–In_2_Se_3_*, (**h**,**i**) a 4 ML *β–In_2_Se_3_* along the $\left[10\bar{1}0\right]$ and $\left[11\bar{2}0\right]$ directions of SiC, respectively. (**j**,**k**) The enlarged RHEED patterns of (**g**,**i**) with enhanced intensity, respectively. The blue curves are the intensity distribution curves of the RHEED patterns.

Figure 1a shows a ball–stick schematic of the crystalline structure of the ML *β–In_2_Se_3_*/BLG heterostructure. According to our RHEED and ARPES results, the lattice orientation of grown In_2_Se_3_ film rotates by ~30° compared to the BLG substrate, as shown in the upper panel of Figure 1a. The stacking order of the five atomic layers of ML *β–In_2_Se_3_* is shown in the middle and lower panels of Figure 1a. Figure 1b,d includes the RHEED patterns of a BLG substrate and a partially covered sub-ML *β–In_2_Se_3_* film along the $\left[10\bar{1}0\right]$ direction of SiC, respectively. The (1 × 1) diffraction stripes of the grown *β–In_2_Se_3_* film nearly coincide with those of the BLG substrate, while, for the RHEED pattern along the $\left[11\bar{2}0\right]$ direction shown in Figure 1e, a new set of diffraction stripes (indicated by the blue arrow) from another direction of the *β–In_2_Se_3_* lattice gradually appeared, which are distinct from the diffraction patterns of the SiC substrate (pointed by the red arrow). The spacing between the (1 × 1) diffraction stripes can be obtained from the intensity distribution curve (yellow dotted line) shown in Figure 1e, from which we can quantitatively determine the in-plane lattice constant of the grown *β–In_2_Se_3_* film as *a* = 4.01 ± 0.05 Å. The detailed method of lattice constant estimation is provided in the Supplementary Materials, Part A. The obtained lattice constant is consistent with its bulk counterpart reported in previous studies [41,55], indicating that the grown *β–In_2_Se_3_* films were almost freestanding with few interfacial strains. Figure 1f,g includes the RHEED patterns of an ML *β–In_2_Se_3_* film along the two directions. When the film fully covered the substrate, the diffraction patterns of the BLG and SiC became totally invisible. Figure 1h,i includes the RHEED patterns of a 4 ML *β–In_2_Se_3_* film along the two directions. As the film thickness increases, the diffraction stripes become slightly sharper. From the distinct features of the (1 × 1) patterns of *β–In_2_Se_3_* along the different directions, we concluded that the lattice orientation of grown *β–In_2_Se_3_* film rotates by ~30° compared to the BLG lattice. More significantly, in the enlarged RHEED images with enhanced intensity shown in Figure 1j,k, the 4 ML one shows a set of weak peaks between the (1 × 1) diffraction stripes, implying a surface reconstruction, while, for the ML one [Figure 1j], no such weak peaks were observed. The surface reconstruction in 4 ML *β–In_2_Se_3_* film was further confirmed in the STM measurements.

**Figure 2:**
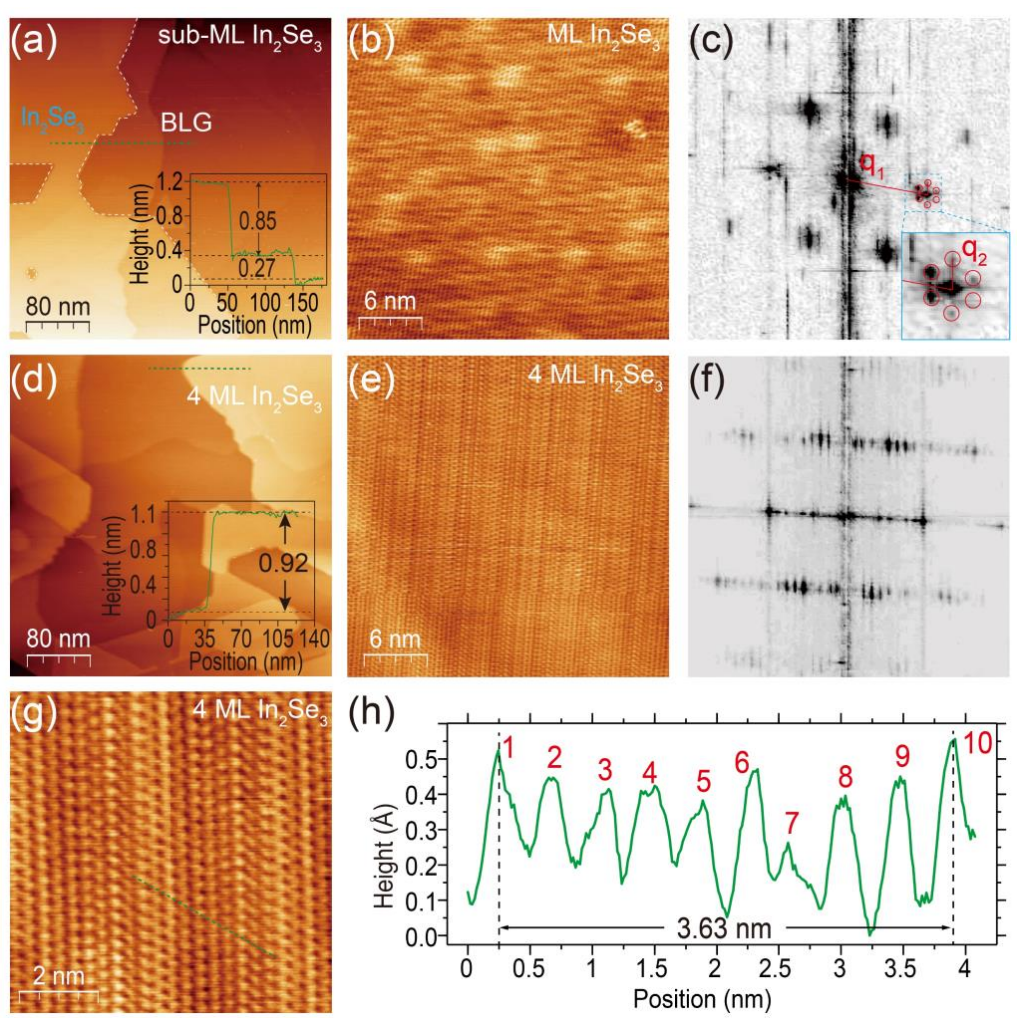
(**a**,**d**) STM topographies of (**a**) sub-ML and (**d**) 4 ML *β–In_2_Se_3_* films, respectively. The insets are the corresponding height profiles along the green dashed lines. (**b**,**c,e,f**) Atom-resolved STM images and the corresponding FFT images on the surface of (**b**,**c**) 1 ML and (**e**,**f**) 4 ML *β–In_2_Se_3_* films, respectively. The inset in (**c**) is a 2× zoom-in of the cray dashed zone. (**g**) Atom-resolved STM image of a 4 ML *β–In_2_Se_3_* film. (**h**) Height profile taken along the green dashed line in (**g**). Scanning parameters: (**a**,**d**) 400 × 400 nm^2^, *V_b_* = 1.00 V, *I_t_* = 100 pA; (**b**,**e**) 30 × 30 nm^2^, *V_b_* = 0.60 V, *I_t_* = 660 pA; (**g**) 8 × 8 nm^2^, *V_b_* = 0.56 V, *I_t_* = 600 pA.

The in situ STM was utilized to investigate the surface topographies and reconstruction of the grown films. In Figure 2a, the STM topography of a sub-ML *β–In_2_Se_3_* film shows that the height of the ML *β–In_2_Se_3_* on BLG is ~0.85 nm, and the height of ~0.27 nm represents the characterized step height of SiC substrate. For the 4 ML *β–In_2_Se_3_* film, the STM image in Figure 2d shows that the height of the top layer *β–In_2_Se_3_* is ~0.92 nm, which is slightly larger than ~0.85 nm of the first ML *β–In_2_Se_3_* grown on BLG, implying that the interfacial interaction between *β–In_2_Se_3_* and graphene layers is slightly stronger than that between *β–In_2_Se_3_* layers itself. In Figure 2b,c, the atom-resolved STM of ML *β–In_2_Se_3_* surface and its fast Fourier transform (FFT) images display the hexagonal symmetry of grown *β–In_2_Se_3_*. In addition, the reciprocal vector $\;\vec{q_{2}}$, displayed in the zoom-in inset of Figure 2b is about 1/7 to the (1 × 1) reciprocal vector $\vec{q_{1}}$, corresponding to the moiré superlattice between ML *β–In_2_Se_3_* and BLG substrate. In Figure 2e,f, the atom-resolved STM and its FFT images of 4 ML *β–In_2_Se_3_* exhibit one-dimensional ferroelectric lattice distortions. To further investigate this structure, we present the zoom-in atom-resolved STM image and its height profile in Figure 2g,h, respectively. We found that this one-dimensional reconstruction exhibits a period of nine peaks, referred to as the (9 × 1) reconstruction phase with a periodical length of ~3.63 nm. Previous research has suggested that this phase is induced by the diploe interaction of the in-plane ferroelectricity of *β–In_2_Se_3_* and can be viewed as a combination of the (4 × 1) and (5 × 1) phases [56,57,58,59]. The (9 × 1) phase is a characteristic reconstruction of the *β*-phase In_2_Se_3_ [58]. Since the one-dimensional distortion on the three-folded rotational symmetric *β–In_2_Se_3_* lattice will have three equivalent orientations, this (9 × 1) reconstruction only appears as a set of weak peaks in the RHEED pattern shown in Figure 1k. We speculate the disappearance of the (9 × 1) distortion in ML *β–In_2_Se_3_* may be attributed to generalized Umklapp scattering induced by the graphene-based superlattice since the wave vector of the ML *β–In_2_Se_3_* is roughly ~$\sqrt{3}$ times the graphene one with an in-plane rotation of ~30° [60,61]. This superlattice will induce the moiré Dirac cone and will be discussed later. All the STM images, together with the sharp RHEED patterns, confirm the high quality of our grown films.

### 3.2. Raman and XPS Characterizations of the Grown β–In_2_Se_3_ Films

**Figure 3:**
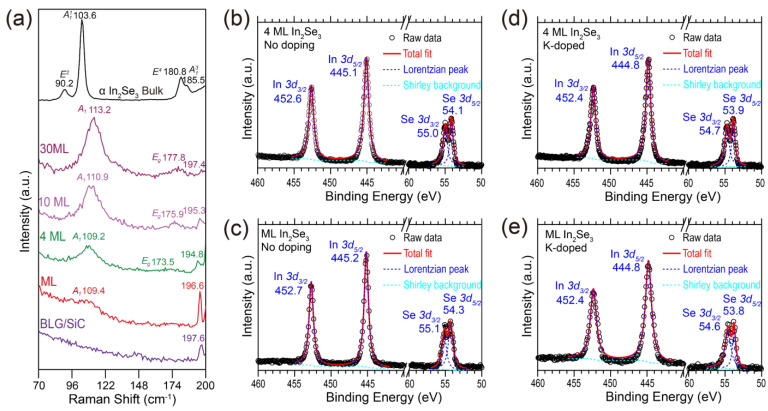
(**a**) Raman spectra of the *α–In2Se3* evaporation source, 30 ML, 10 ML, 4 ML, ML *β–In_2_Se_3_* films, and BLG/SiC substrate. (**b**–**e**) XPS spectra of In 3*d*_3/2_, In 3*d*_5/2_, Se 3*d*_3/2_, and Se 3*d*_5/2_ orbitals of (**b**) 4 ML, (**c**) 1 ML, (**d**) potassium-doped 4 ML, and (**e**) potassium-doped ML *β–In_2_Se_3_* films, respectively. The red lines are the multiple Lorentzian peaks fitting to the raw data by using Equation (1). The blue dashed lines are the parts of Lorentzian peaks of the total fitting lines. The cyan dashed lines are the Shirley backgrounds of the raw data.

To further evidence the structural phase of the grown In_2_Se_3_ films, we conducted the ex situ Raman measurements on the grown *β–In_2_Se_3_* films and the evaporation source *α–In2Se3*. The top black line in Figure 3a represents the Raman spectrum of the bulk *α–In2Se3* source material, and its characteristic peaks around 90.2, 103.6, 180.8, and 185.5 cm^−1^ correspond to the *E*^2^, $A_{1}^{1}$, *E*^4^, and $A_{1}^{3}$ modes, respectively [62]. For the four curves plotted below, which are the Raman spectra of 30 ML, 10 ML, 4 ML, and ML *β–In_2_Se_3_* films, the characteristic peaks of *α–In2Se3* (*E*^2^ mode) were not observed. Instead, the *A*_1_ mode around 110 cm^−1^ and *E_g_* mode between 173.5 and 177.8 cm^−1^ emerged, indicating the pure *β-*phase of grown In_2_Se_3_ films [39]. In addition, the full-width-at-half-maximum (FWHM) of *β–In2Se3 A*_1_ mode is larger than that of *α–In2Se3*$\;A_{1}^{1}$ mode, which is consistent with the previous report [62]. The 197 cm^−1^ peaks in the Raman spectra were all from the 4H-SiC substrate [63]. The above Raman spectral features are sufficient to confirm that the grown film is in the *β* phase rather than *α* phase.

**Figure 4:**
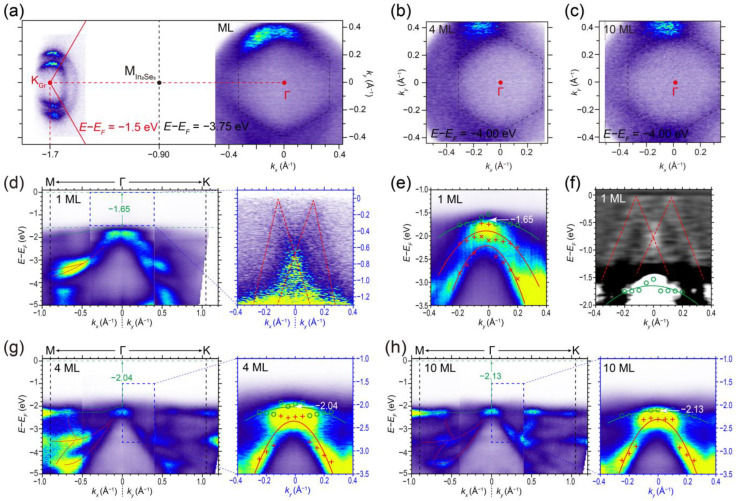
(**a**–**c**) Constant energy mappings of (**a**) sub-ML, (**b**) 4 ML, and (**c**) 10 ML *β–In_2_Se_3_* films, respectively. (**d**) ARPES spectra of the ML *β–In_2_Se_3_*/BLG along the M-Γ-K direction. The right panel is the zoom-in spectra of the blue rectangular region. (**e**) Zoom-in ARPES spectra around the VBM of ML *β–In_2_Se_3_*. (**f**) Second-derivative ARPES spectra of ML *β–In_2_Se_3_*. (**g**,**h**) ARPES spectra of the 4 and 10 ML *β–In_2_Se_3_* films along the M-Γ-K direction, respectively. The right panels are the zoom-in spectra of the blue rectangular regions.

Figure 3b,c shows the XPS spectra around In 3*d* and Se 3*d* orbitals for the 4 and 1 ML *β–In_2_Se_3_* films, respectively. To better obtain the core levels, we completed the multiple Lorentzian peaks fitting on the raw data by using the following multiple Lorentzian peaks fitting equation:

(1) $$I\left(E_{b}\right)=S\left(E_{b}\right)+\overset{n}{\underset{i=1}{\sum }}\frac{A_{i}}{1+\frac{{\left(E_{b}-E_{i}\right)}^{2}}{W_{i}^{2}}}$$

while $I\left(E_{b}\right)$ is the intensity of XPS spectrum, $E_{b}$ is the binding energy, $S\left(E_{b}\right)$ is the Shirley background [64,65], n is the number of peaks, $A_{i}$ is the height of each peak, $E_{i}$ is the peak position of each peak, and $2W_{i}$ is the FWHM of each peak. The total fitting lines are plotted as the red lines, and each peak is plotted as the blue dashed lines. The core levels of In 3*d*_3/2_ (452.6 eV), In 3*d*_5/2_ (455.1 eV), Se 3*d*_3/2_ (55.0 eV), and Se 3*d*_5/2_ (54.1 eV) orbitals of the 4 ML *β–In_2_Se_3_* film exhibit small redshifts of about 0.1~0.2 eV compared to those of In 3*d*_3/2_ (452.7 eV), In 3*d*_5/2_ (455.2 eV), Se 3*d*_3/2_ (55.1 eV), and Se 3*d*_5/2_ (54.3 eV) orbitals in ML *β–In_2_Se_3_* film. The binding energy shifts are likely due to the charge transfer effect between the BLG substrate and ML *β–In_2_Se_3_*, as indicated by the different energy positions of the VBM between 1 and 4 ML *β–In_2_Se_3_* in Figure 4. For the XPS spectra of potassium-doped 4 and 1 ML *β–In_2_Se_3_* films shown in Figure 3b,c, the core levels exhibit redshifts of about 0.3~0.5 eV compared to the pristine *β–In_2_Se_3_* films. Unfortunately, since the XPS signal of potassium *2p* orbital is mixed with the Se Auger line L_3_M_23_M_45_(^1^P) [66,67] and the amount of potassium dopant is rather small, it is very difficult to distinguish the rather weak potassium signal in the XPS spectra (see Supplementary Materials, Part B).

### 3.3. Band Structures Evolution of β–In_2_Se_3_ Films

We further investigated the energy band evolution of *β–In_2_Se_3_* films with increasing thickness and surface doping effect via in situ ARPES. To identify the high symmetry points in reciprocal space, we plotted the constant energy ARPES mapping of the ML *β–In_2_Se_3_* in Figure 4a. To indicate the 30° relative rotation angle of the BLG substrate and ML *β–In_2_Se_3_*, the mapping energies of them in Figure 4a are various for different momentum positions. The constant energy mapping around the K point of graphene Brillouin zone, taken at *E* − *E_F_* = −1.50 eV, shows the characterized Dirac cone pockets of BLG substrate, and the red solid line denotes the Brillouin zone boundary of graphene. The Dirac point of BLG substrate is located at −0.30 eV below Fermi level with no energy shift (Supplementary Materials, Figure S3) [52,68], indicating no-charge doping from the defects of substrate. The mapping taken at *E* − *E_F_* = −3.75 eV around the Γ point represents a hole band pocket of ML *β–In_2_Se_3_* (denoted by the black dashed hexagon). The black dotted line at *k* = − 0.905 Å^−1^ represents the *β–In_2_Se_3_* Brillouin zone boundary. Figure 4b,c includes the constant energy mapping of 4 and 10 ML *β–In_2_Se_3_* films taken at *E* − *E_F_* = −4.00 eV, respectively. The symmetry of the hole band pocket remains unchanged with the increasing thickness of *β–In_2_Se_3_*.

Figure 4d is the energy–momentum ARPES cut of the ML *β–In_2_Se_3_*/BLG along the M-Γ-K direction. The right panel is the zoom-in spectra with enhanced intensity near Fermi level, in which a weak Dirac cone (depicted by the red dashed lines) emerges at Γ point. This weak Dirac cone was not observed in the 4 and 10 ML *β–In_2_Se_3_* films. The second derivative spectra in Figure 4f imply that this weak Dirac cone at Γ point has the characteristics of epitaxial graphene on SiC [60,69], which can be attributed to the Umklapp scattering in the *β–In_2_Se_3_*/BLG heterostructure [60]. This emergence of renormalized moiré Dirac cones suppresses the formation of (9 × 1) surface reconstruction and makes the ML *β–In_2_Se_3_*/BLG heterostructure a semi-metal, which contrasts with the semiconductive multilayer *β–In_2_Se_3_* films [Figure 4g,h].

Now, we focus on the valence bands of *β–In_2_Se_3_* below the scattering-induced Dirac cone. The ML *β–In_2_Se_3_* ARPES spectra in Figure 4e along with its second-derivative spectra in Figure 4f show that the VBM is located at the Γ point. For the 4 and 10 ML *β–In_2_Se_3_* films, the momentum position of VBM remains unchanged at the Γ point, as shown in Figure 4g,h. The VBM of ML *β–In_2_Se_3_* is located at −1.65 eV below the Fermi level. However, the VBMs of the 4 and 10 ML *β–In_2_Se_3_* are located at −2.04 eV and −2.13 eV, respectively. The higher VBM of ML *β–In_2_Se_3_* compared to the other multilayer films is due to the charge transfer effect and the moiré superlattice between the *β–In_2_Se_3_* and BLG substrate. The energy positions of VBM are determined by parabolic fitting of the valence band data, which are extracted from the fitting of the energy distribution curves (EDCs) in Figure S2. The valence band data are plotted as the colored circles/crosses/forks in Figure 4e and the right panels of Figure 4g,h, and the parabolic fitting results are plotted by the colored curves. The detailed method of the VBM determination is provided in the Supplementary Materials, Part D. The deep valence bands ranging from −5.00 eV to −2.50 eV are depicted by the red lines in Figure 4d,g,h, showing distinct features for different thicknesses of *β–In_2_Se_3_* films.

**Figure 5:**
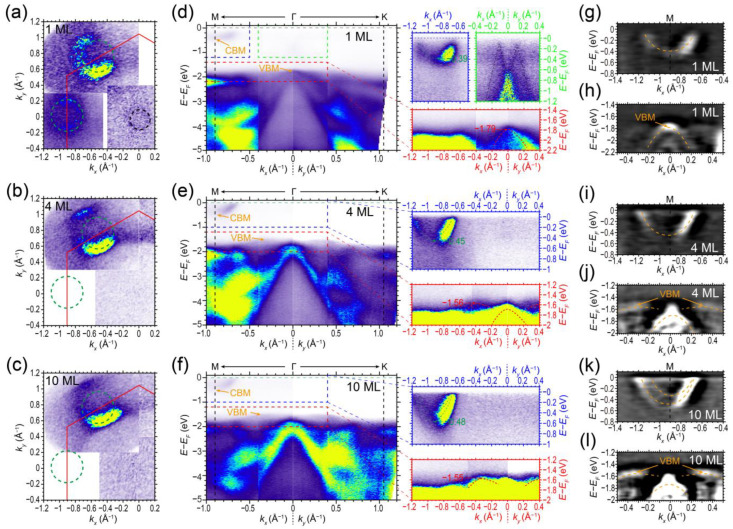
(**a**–**c**) Fermi surface mappings of the (**a**) 1, (**b**) 4, and (**c**) 10 ML *β–In_2_Se_3_* films with potassium doping, respectively. (**d**–**f**) ARPES spectra of (**d**) 1, (**e**) 4, (**f**) 10 ML *β–In_2_Se_3_* films with potassium doping along the M-Γ-K direction, respectively. The right panels are the zoom-in spectra with enhanced intensity in the colored rectangular regions. (**g**–**l**) Second-derivative ARPES spectra of the (**g**,**h**) 1, (**i**,**j**) 4, and (**k**,**l**) 10 ML *β–In_2_Se_3_* films around the CBM and VBM, respectively.

In order to observe the conduction band of the grown *β–In_2_Se_3_* films, we doped the film surface by potassium. This doping process can lift the Fermi level upward and allow the conduction band minimum (CBM) to be accessible for ARPES measurements. Figure 5 shows the ARPES spectra of the 1, 4, and 10 ML *β–In_2_Se_3_* films after potassium doping with the same dosage. Figure 5a–c includes the Fermi surface mappings of 1, 4, and 10 ML *β–In_2_Se_3_* films with potassium doping, respectively. The hexagonal electron pockets depicted by the green dashed circles are visible around the M point at the Brillouin zone boundary, consistent with the previous experiments on bulk *β–In_2_Se_3_* [32,55,70]. Additionally, we also found a small pocket (depicted by the black dashed circles) at the Γ point in ML *β–In_2_Se_3_*, which originates from the moiré Dirac cone from ML *β–In_2_Se_3_*/BLG hetero-interface.

Figure 5d–f shows the energy–momentum ARPES spectra of 1, 4, and 10 ML *β–In_2_Se_3_* films with potassium doping along the M-Γ-K direction, respectively. The zoom-in spectra with enhanced intensity are at the right panels with corresponding colored axis. The moiré Dirac cone at the Γ point can be clearly observed in the zoom-in spectra of ML *β–In_2_Se_3_* film but disappears for the 4 and 10 ML *β–In_2_Se_3_* films. The conduction band along the Γ-M direction was found below Fermi level after potassium doping, with the momentum position of the CBM located at the M point of the Brillouin zone (*k_M_* = ~0.905 Å^−1^). The CBM of potassium-doped ML *β–In_2_Se_3_* film is at −0.38 eV below the Fermi level. For the potassium-doped 4 and 10 ML *β–In_2_Se_3_* films, the CBMs are located at ~−0.45 eV and −0.48 eV, respectively. The evolution of the conduction band structures with increasing thickness can be more clearly revealed in the second-derivative spectra shown in Figure 5g,i,k. In contrast to the single conduction band observed in 1 and 4 ML *β–In_2_Se_3_*, the conduction band of the 10 ML *β–In_2_Se_3_* in Figure 5k displays a splitting into two branches (depicted by the orange dashed curves). For the n-type doping bulk situation in a previous report [70], the conduction band also splits into several branches. Here, the 10 ML *β–In_2_Se_3_* behaves as the bulk situation with split conduction band.

The valence bands of the ML *β–In_2_Se_3_* show similar behaviors to its conduction bands after potassium doping. In Figure 5d, the VBM of the ML *β–In_2_Se_3_* film shifts downwards to −1.79 eV, or by 0.14 eV compared to the pristine film. In contrast, for the 4 and 10 ML *β–In_2_Se_3_* films, the VBM shifts upwards to −1.56 eV and −1.55 eV, respectively. Compared to the pristine films, the VBM shifts upwards by 0.48 eV and 0.58 eV for 4 ML and 10 ML *β–In_2_Se_3_* films, respectively. This means that the VBM of the multilayer *β–In_2_Se_3_* films was elevated after potassium doping. Given the energy positions of CBM, the indirect band gap of *β–In_2_Se_3_* films after potassium doping was estimated to be 1.40 eV, 1.11 eV, and 1.07 eV for the 1, 4, and 10 ML *β–In_2_Se_3_* films, respectively. This indicates that the band gaps shrink by at least 0.25 eV, 0.93 eV, and 1.06 eV for the 1, 4, and 10 ML *β–In_2_Se_3_* films, respectively. The shrinkage of bandgap after potassium doping would be the reason for relatively less downshift and even upshift regarding VBM towards the Fermi level by potassium doping.

By comparing ARPES and XPS results on the potassium-doped *β–In_2_Se_3_* films, we found that the potassium doping leads to different energy shifts of core levels and VBM for different thicknesses. The shifts in core levels are all about 0.3~0.5 eV towards lower binding energy, which is generally consistent with the energy shift of VBM for 4 ML *β–In_2_Se_3_* films, while, for the ML *β–In_2_Se_3_* films, the VBM shifts to higher binding energy by 0.14 eV, which is opposite to the shifts of core levels. This difference may be attributed to the interfacial effects of ML *β–In_2_Se_3_*/BLG heterointerface.

In addition to the gap shrinkage caused by potassium doping, the momentum positions of the VBM of 4 and 10 ML *β–In_2_Se_3_* also shift away from the Γ point after doping. From the enhanced intensity spectra (bottom-right panels of Figure 5d–f) and the second-derivative spectra (Figure 5h,j,l) of 1, 4, and 10 ML *β–In_2_Se_3_* films, we found that the momentum positions of VBM of 4 and 10 ML *β–In_2_Se_3_* films shift to *k_VBM_* ≈ ±0.35 Å^−1^ (labeled by the orange arrows). However, the VBM of ML *β–In_2_Se_3_* remained at the Γ point. This can be attributed to the fact that the bands at different momentum positions in reciprocal space have different responses to the surface doping, as observed in previous reports [27,71]. Although the momentum positions of VBM were moved by potassium doping, the 4 and 10 ML *β–In_2_Se_3_* films were still indirect semiconductors.

## 4. Conclusions

In summary, we have successfully realized the MBE growth of *β–In_2_Se_3_* thin films on BLG substrates by using *α–In2Se3* and Se shots serving as the evaporation sources. We found that the lattice orientation of grown *β–In_2_Se_3_* rotates by ~30° compared to the BLG substrate. The 4 ML In_2_Se_3_ film shows a characterized (9 × 1) reconstruction of *β–In_2_Se_3_*, while the ML In_2_Se_3_ shows no surface reconstruction due to the interfacial interaction and moiré superlattice between ML *β–In_2_Se_3_* and BLG substrate. The interfacial moiré modulation results in a folding Dirac cone structure at the Γ point in the ML *β–In_2_Se_3_*/BLG heterostructure. In addition, we found that the band gap of *β–In_2_Se_3_* films shrinks after potassium doping. For the 4 and 10 ML *β–In_2_Se_3_* films with potassium doping, the momentum positions of VBM move away from the Γ point along the Γ-M direction. Our work provides inspiration for the synthesis and electronic characterization of the epitaxial In_2_Se_3_ films in 2D limit, which would be a new platform for studying the 2D ferroelectric heterostructures and devices. The high quality of the grown films would also provide an ideal platform to fabricate 2D heterostructures; for instance, some interesting 2D materials, such as TMDCs, could be grown on its surface to realize the band engineering of semiconductors, which has been theoretically proposed in a previous study [11]. Additionally, the growth of pure-phase *β–In_2_Se_3_* films by using *α–In2Se3* shots would fulfill the phase diagram of In_2_Se_3_ synthesis.

## Data Availability

The data presented in this study are available on request from the corresponding author.

## Supplementary Materials

The following supporting information can be downloaded at: https://www.mdpi.com/article/10.3390/nano13091533/s1, lattice constant determined from the RHEED patterns (Figure S1), XPS spectra of potassium dopant (Figure S2), ARPES spectra of BLG substrate (Figure S3) determination of the VBM from ARPES spectra (Figure S4). References [52,66,67,68] are cited in the supplementary materials.
