# Supplementary material for: Thickness-Dependent Evolutions of Surface Reconstruction and Band Structures in Epitaxial β–In2Se3 Thin Films
Source: Nanomaterials (Basel). 2023 May 3;13(9):1533. doi: 10.3390/nano13091533 (PMC10180126; doi:10.3390/nano13091533)
Supplement: Supplementary file 1 [file nanomaterials-13-01533-s001.zip › nanomaterials-2364183-supplementary.pdf]

Supplementary Materials for

Thickness-dependent Evolutions of Surface Reconstruction and Band Structures in Epitaxial β-In_2Se_3 Thin Films

Qinghao Meng, Fan Yu, Gan Liu, Junyu Zong, Qichao Tian, Kaili Wang, Xiaodong Qiu, Can Wang , Xiaoxiang Xi , Yi Zhang

A. Lattice constant determined from the RHEED patterns

For the non-fully covered sub-ML ***β***-In_2Se_3 film, we can simultaneously observe the diffraction pattern from both the substrate BLG/SiC and the grown ***β***-In_2Se_3 film. Thus, we can determine the lattice constant of the grown ***β***-In_2Se_3 film by comparing the spacings of the 1×1 diffraction spots between the SiC substrate and the ***β***-In_2Se_3 film.

Figure S1(a) show a typical non-fully covered ML ***β***-In_2Se_3 film. We can extract the intensity distribution line of the yellow rectangle region along the horizontal direction [see Figure S1(b)]. Then, we can get the spacing of the 1×1 diffraction spots of the SiC substrate (L_{SiC}) and the spacing of the 1×1 diffraction stripes of the grown ***β***-In_2Se_3 film (L_{In2Se3}). The lattice constant of the grown ***β***-In_2Se_3

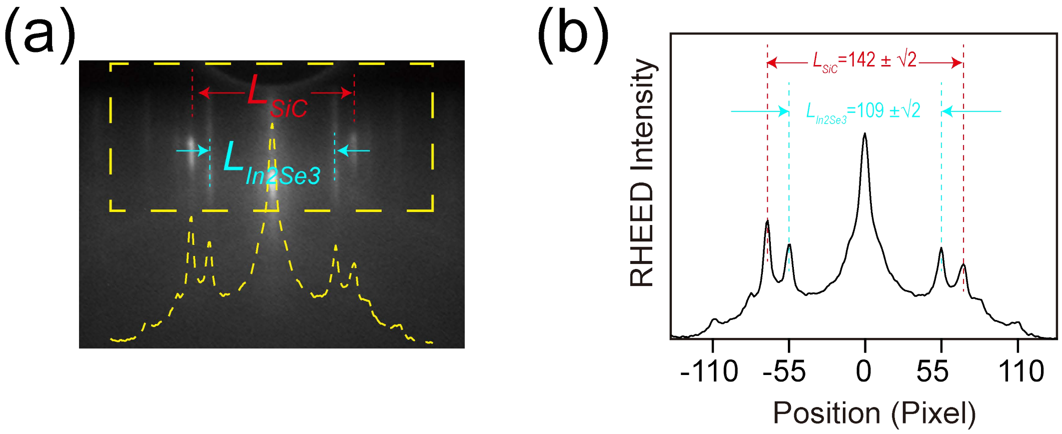

Figure S1 (a) RHEED pattern of a not-fully covered ML ***β***-In_2Se_3 film on BLG/SiC substrate along the $[11\bar{2}0]$ direction. **(b)** RHEED intensity distribution curve of the yellow rectangle region in (a) along the horizontal direction.

film ($\boldsymbol{a}_{\mathrm{In2Se3}}$) can be written as: $\boldsymbol{a}_{\mathrm{In2Se3}} = \frac{L_{SiC}}{L_{In2Se3}} \times 3.08$ Å, where the 3.08 Å is the known lattice constant of the SiC substrate. The resolution limit is mainly determined by the resolution ability of the camera system. Here we estimate the spacing error to be $\sqrt{2}$ pixel of the RHEED image taken by the camera system, and the error of lattice constant should be $\frac{\sqrt{2}}{L_{\mathrm{In2Se3}}} \times \boldsymbol{a}_{\mathrm{In2Se3}}$ Å. Then we get the in-plane lattice constant of $\boldsymbol{\beta}$-In_2Se_3 as $\boldsymbol{a}_{\mathrm{In2Se}} = 4.01 \pm 0.05$ Å.

B. XPS spectra of potassium dopant

Figure S2 is the XPS spectra of the ML and 4 ML ***β***-In_2Se_3 films with and without potassium doping. The XPS signal of potassium *2p* orbital is mixed with the Se Auger line $L_3M_{23}M_{45}(^1P)$,[1,2] and is rather weak, thus is difficult to distinguished from the Se Auger peak. The rather we weak signal of potassium indicates the small amount of potassium dopant on the ***β***-In_2Se_3 surface.

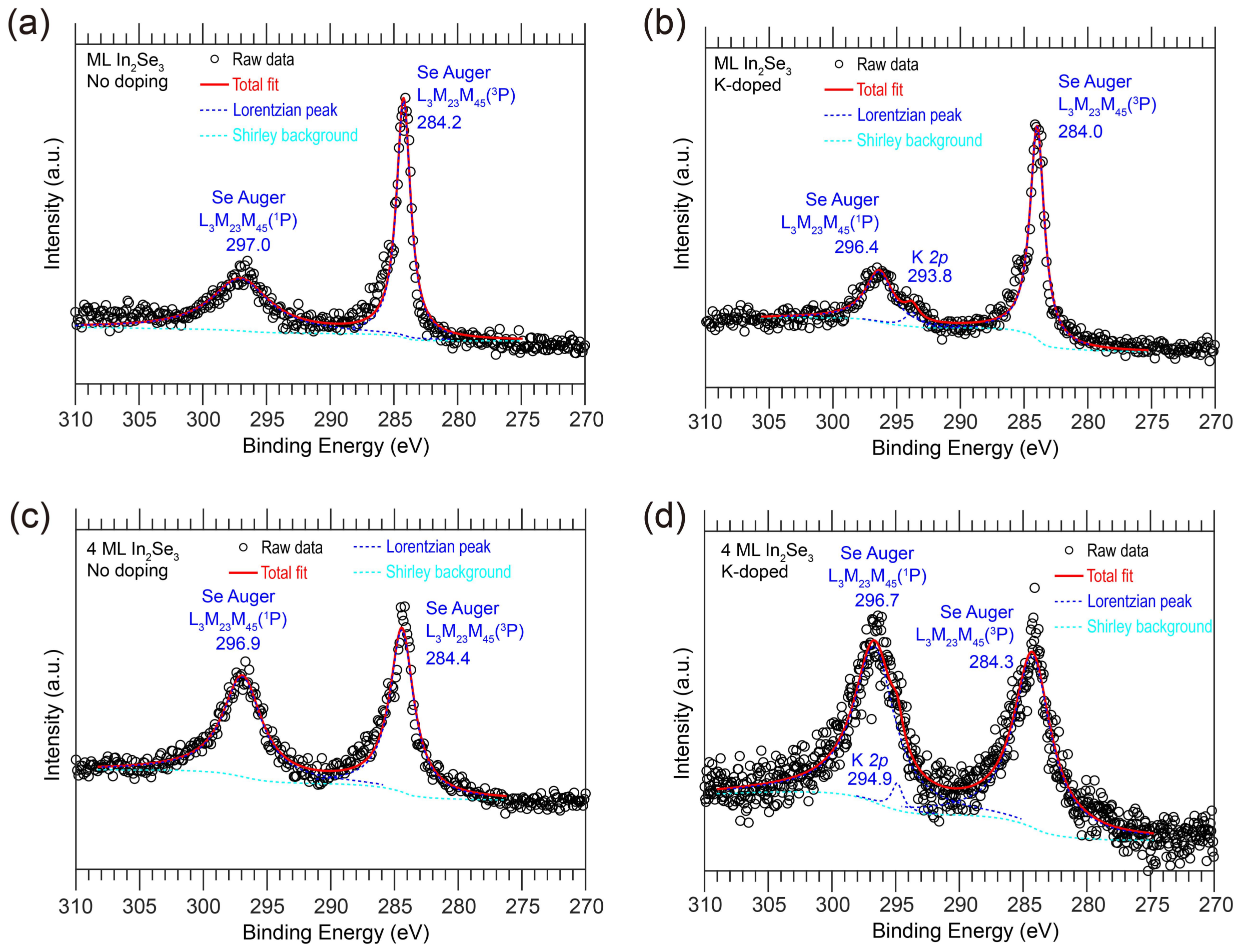

Figure S2 (a) & (b) XPS spectra of (a) pristine and (b) potassium-doped ML ***β***-In_2Se_3 films, respectively. **(c) & (d)** XPS spectra of (c) pristine and (d) potassium-doped 4 ML ***β***-In_2Se_3 films, respectively. The red lines are the multiple Lorentzian peaks fittings of the raw data. The blue dashed lines are the parts of Lorentzian peaks. The cyan dashed lines are the Shirley background.

C. ARPES spectra of BLG substrate

Figure S3 is the ARPES cut of BLG substrate taken at the K point of Brillouin zone. The Dirac point of BLG is located at −0.30 eV below Fermi level with no energy shift,[3,4] indicating no charge doping from the defects of substrate.

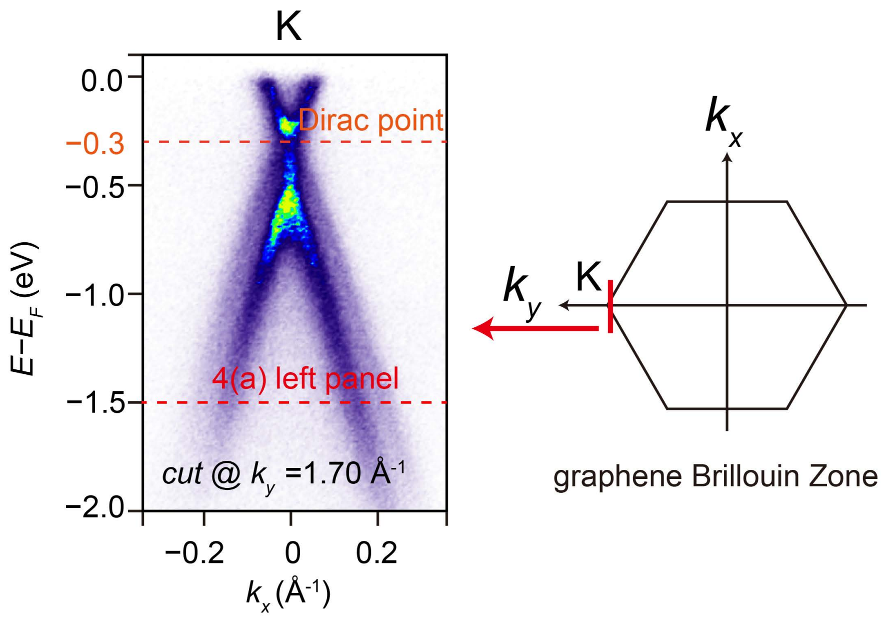

Figure S3 APRES cut of BLG substrate taken at the K point of Brillouin zone.

D. Determination of the VBM from ARPES spectra

Figures S4(a)-4(c) show the energy distribution curves (EDCs) of the ARPES spectra taken around the VBM as shown in Figures S4(d)-4(f), which is corresponding to the 1, 4 and 10 ML ***β***-In_2Se_3 films from energy range of −3.5 to −1 eV and reciprocal momentum vector range of $\boldsymbol{k}_y = \pm 0.20$ Å^{-1}, respectively. For each EDC, we use following multiple peaks function to fit experimental curve,

$$I(E) = P_0 + P_1 \cdot E + \sum_{i=1}^{n} \frac{P_{ia}}{1 + \frac{(E - P_{ix})^2}{P_{iw}^2}} \qquad (1)$$

where the $P_0 + P_1 \cdot E$ is the linear background, the $\sum_{i=1}^{n} \frac{P_{ia}}{1+\frac{(E-P_{ix})^2}{P_{iw}^2}}$ is the multiple Lorentzian peak function, n the number of peaks, P_{ia} the peak height, P_{iw} the peak width, P_{ix} the peak position. For

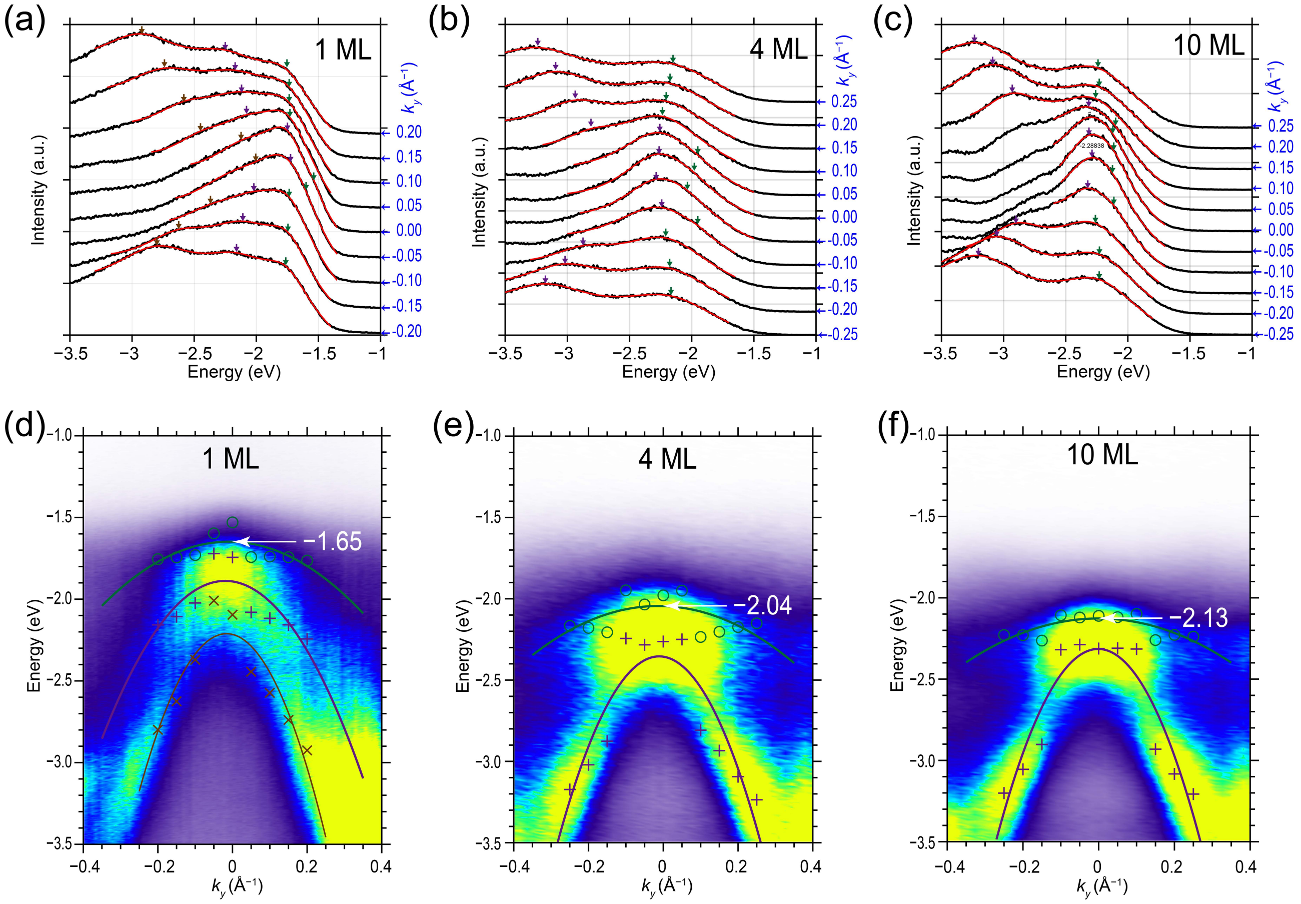

Figure S4 (a) – (c) The energy density curves (EDCs) stacking of the ARPES data momentum cuts from **(a)** ML ***β***-In_2Se_3/BLG heterostructure, **(b)** 4ML ***β***-In_2Se_3 films and **(c)** 10ML ***β***-In_2Se_3 films on BLG substrate near the Γ point of the Brillouin Zone. The valence bands nearest to the Fermi level are labeled as yellow lines

ML $\boldsymbol{\beta}$-In_2Se_3 EDCs, $n = 3$ was applied since three peaks can be resolved from the EDCs. For 4 and 10 ML $\boldsymbol{\beta}$-In_2Se_3 EDCs, $n = 2$ was applied since only two peaks can be resolved from the EDCs. The fitting results were plotted as the red curves in Figures S4(a)-4(c), consistent well with the experimental curve. The fitting results of peak positions were marked by the colored arrows in in Figures S4(a)-4(c), and were plotted as the colored circles/crosses/forks in Figures S4(d)- 4(f). Then, we used the parabolic fitting to the peak positions data (band data) shown in Figures S4(d)-4(f). The fitting results were plotted as the colored curves in Figures S4(d)-4(f). The energy positions of VBM can be extracted from the parabolic fitting curves and were labeled by the white arrows and texts in in Figures S4(d)-4(f). The fitting errors of the VBM is about ±30 meV in average.

References

1 John F. Moulder, William F. Stickle, Peter E. Sobol et al., *Handbook of X-ray Photoelectron Spectroscopy*. (Perkin-Elmer Corporation, Physical Electronics Division, United States of America, 1992).

2 P. Weightman, E. D. Roberts, and C. E. Johnson, L2,3MM Auger processes in selenium *Journal of Physics C: Solid State Physics* **8** (4), 550 (1975).

3 Y. Qi, S. H. Rhim, G. F. Sun et al., Epitaxial Graphene on SiC(0001): More than Just Honeycombs *Physical Review Letters* **105** (8), 085502 (2010).

4 E. Moreau, S. Godey, F. J. Ferrer et al., Graphene growth by molecular beam epitaxy on the carbon-face of SiC *Applied Physics Letters* **97** (24), 241907 (2010).
